# Exploring Extracurricular Clubs for Building Social Competence of Students With Autism

**DOI:** 10.3389/fpsyt.2022.840294

**Published:** 2022-03-23

**Authors:** Sara L. McDaniel, Laura J. Hall, Bonnie K. Kraemer

**Affiliations:** ^1^Department of Educational Psychology, California State University, East Bay, Hayward, CA, United States; ^2^Department of Special Education, San Diego State University, San Diego, CA, United States

**Keywords:** autism, high-functioning autism (HFA), social communication, 21st century skills, extracurricular clubs, high school, mixed methods, inclusive settings

## Abstract

Individuals with autism experience challenges in social communication that directly impacts in-school and post-school performance. A growing number of these students are taught in general education settings in public high schools, where creating opportunities for practice of social communication skills is frequently a challenge. This exploratory, mixed methods pilot investigation explores existing and potential opportunities for high school students with autism to practice 21st century skills, including communication, in extracurricular club environments. Findings indicate that extracurricular club settings are rich environments in which all participating students, including those with autism, have opportunities to practice critical 21st century skills in a context related to their interests.

## Introduction

As of the 2018/2019 school year, there were approximately 762,000 students receiving special education services under the disability category of autism of the Individuals with Disabilities Education Improvement Act (1), equating to 1.5% of total population of students enrolled in schools in the United States. This calculation is not inclusive of the additional students with autism who receive services under other categories (e.g., Other Health Impairment) or who do not receive special education services. Over half of children identified with autism possess average or above average intellectual ability (2). The worldwide trend is toward the inclusion of students with disabilities in school environments, which requires providing students supported access to all academic and social settings (3). Of students with autism in U.S. public schools, 18.5% spend between 40 and 79% of their day in general education settings, and 40 spend 80% or more of their day in general education settings U.S. Department of Education, National Center for Education Statistics (1).

Even when students are taught in general education classrooms, opportunities for peer interaction and to practice social skills may be infrequent. This opportunity is important because individuals with autism can experience difficulties in the area of social communication that interfere with their ability to communicate or relate to other people [5^th^ ed, (4)], and social competence is one of the key factors that results in job loss after high school (5). The most recent data available indicate that only 58% of young adults with autism report working for pay after high school, with most employed in part-time or low-wage positions, while only 36% receive any type of postsecondary education and just one in five live independently (6). Results such as these make it clear that high schools are failing to meet the unique needs of students with autism. A focus on building social communication skills is crucial to preparing these young people for productive and fulfilling lives post-high school.

The differences in the quality and frequency of peer-to-peer interactions experienced by individuals with autism can lead them to have less-than-fulfilling social experiences and hinder their development of social communication skills (7). Peer-mediated instruction and intervention, which is an evidence-based approach for students with autism (8) may exist in some high schools (i.e., peer buddy programs), but students with autism who spend most of their time in general education may not have access to these programs. Due to complex diploma and graduation requirements, high school students' schedules are often too full to make room for social skills interventions, and social skills interventions are not typically part of the curriculum for diploma-bound students with autism (9).

Extracurricular clubs and activities may serve as a context to address these unmet social needs. Several studies have recommended incorporating students' interests into activities in order to increase their motivation to participate (10, 11), and suggested that activities based on preferred interests improve socialization with typical peers (12, 13). Participation in extracurricular clubs is one of the recommended activities in the school-based PEERS^®^ curriculum (14) due to the typical opportunities to engagei n peer interaction.

Agran et al. (15) found that extracurricular activities represent an under-utilized opportunity to support the social communication development of students with autism. Conveniently, most high schools already offer clubs designed around student interests. Previous research (16, 17) indicated that participation in extracurricular clubs fostered peer relationships, increased a sense of belonging within the school community, and helped students develop important social, academic, leisure, and life skills. Such clubs were found to be natural settings in which students could practice skills within applied contexts (11, 15). Despite the fact that special education professionals have for decades been recommending extracurricular activities for students with disabilities, an analysis of over 400 students' individualized education plans (IEPs) found that <12% included any reference to extracurricular activities (18).

Agran et al. (15) surveyed 143 special educators across five U.S. states about their students' participation in extracurricular activities. A large majority of these teachers (62%) reported that students rarely participated in school-based extracurricular activities, and nearly half (47%) reported that the students they worked with rarely expressed interest in becoming involved with such activities. Notably, the opportunity to practice social communication and functional skills was most commonly ranked as the most important benefit associated with participating in extracurricular activities.

Addressing college and career readiness skills of all students is a focus for many high schools. The Framework for 21st Century Learning, compiled by the Partnership for 21st Century Learning (a Network of Battelle for Kids), specifies the skills high school graduates need to master in order to be prepared for college or careers based on input collected from educators, education experts, and business leaders (19). This framework includes a Learning and Innovation Skills pillar, which focuses on *critical thinking, collaboration, communication*, and *creativity*, or “the 4Cs.” These 4Cs align with areas in which students with autism may struggle and are a critical focus of improving the poor post-school outcomes for students with autism. Specifically, collaboration and communication skills are required to understand and respond appropriately in social and workplace situations, and such demands are often difficult for people with autism (20). Extracurricular clubs have the potential to be a context in which all students have the opportunity to practice skills that enable them to participate fully in society.

The purpose of this pilot study was to obtain perspectives from stakeholders in order to describe extracurricular club settings and determine the opportunities that currently exist for students, including those with autism, to focus on 21st century skills. This exploratory, mixed-methods pilot study extends the work by Agran et al. (15) in two important ways: (1) by including not only self-report survey data from special educators, but also student interviews supplemented by observations of students in club settings, and (2) by focusing on the experiences of students with autism who are educated exclusively or nearly exclusively in general education settings. This investigation was completed using participants from a larger RCT conducted by the *Center on Secondary Education for Students with Autism Spectrum Disorders* (CSESA) (21). As is recommended when designing feasible and comprehensive school-based interventions, care was taken to ensure that stakeholders who could provide relevant opinions and thoughts on this topic were included (22). The following questions guided this research:

1. What is the content of extracurricular high school clubs that attracts student participants with autism, and what is the mission or goal of these clubs?
2. How do students get involved in extracurricular clubs on high school campuses, and are there barriers to participation for students with autism?
3. Do students with autism have opportunities for social interaction with peers during extracurricular clubs?
4. Do participants in high school clubs have the opportunity to work on the 4Cs of 21st century skills in these clubs? If not, what are the barriers?
5. What do students with autism report about their experiences in high school clubs, including the experiences they value, the aspects they identify as challenges, and their preferences for peer interaction?

## Methods

This was an exploratory mixed-methods pilot study with multiple sources of data, including a survey of special education teachers, semi-structured interviews of high school students with autism, and observations of high school students with autism in club environments. Concurrent triangulation was used to validate the findings generated from each data source with evidence produced by the others, with this data integration ultimately providing comprehensive results (23, 24).

### Participants

All participants in the present study were originally selected as part of a nationwide randomized controlled trial (RCT) investigation by CSESA of a comprehensive multi-component intervention for high school students with autism [for more information related to the CSESA sampling procedure and demographic information related to the sample, see (21)]. Participants in this study were a sample of convenience, recruited from the greater CSESA study sample at one of the three regional sites, which included 20 public high schools, and represent two major stakeholder groups: (1) special educators serving students with autism, and (2) students with autism who participated in extracurricular clubs. The students with autism who participated in CSESA included both those who were diploma-bound and those earning certificates of completion via an alternative curriculum.

#### Special Educators

All participating special educators were members of their site-wide CSESA teams at one regional site. These teachers worked closely with students with autism and had knowledge about the participation of their students in extracurricular activities on campus, and were invited to participate in the study via survey. Survey respondents (*N* = 34) had an average of 11 years' experience working with students with autism ranging from 2 to 28 years. Seven (21%) of the special educators had students with autism on their caseload who spent 80% or more of their day in general education, while 27 (79%) of the special educators had students with autism who spent between 40 and 79% of their day in general education.

#### Students With Autism

A convenience sample of CSESA students (*N* = 8) who met all three of the following criteria for selection were included in the study: (1) the student was expected to earn a high school diploma, rather than a certificate of completion; (2) the student had an intellectual ability in the average to above average range, and (3) the student participated in at least one extracurricular club at their high school. Each school involved in CSESA appointed a site coordinator, and these individuals provided information regarding the club participation of CSESA students at their site. These students provided assent for participation in the interview and/or observational components of this study. Six provided assent for observation during club activities, and six provided assent to be interviewed (five assented to both). 6. The student sample consisted of six male (Harry, Luke, Tyrese, Steven, Jesus, and Roberto) and two female (Courtney and Stefana) students between the ages of 16 and 18 who participated in eight different extracurricular clubs. Of these students, four spent 67% of their day in general education, one spent 83% of his day in general education, and three spent 100% of their day in general education. Table 1 provides a description of the student participants, including the extent of their participation in the study and in their clubs.

**Table 1:** Student participants.

**Name**	**Age**	**Gender**	**Club**	**Role**	**% in general education**
Harry^*^^~^	17	M	Stage crew club	Member	100%
Courtney^*^^~^	16	F	Girls learn international	Member	67%
Luke^*^^~^	18	M	Video game club	Founder/President	67%
Tyrese^*^^~^	16	M	Movie club	Founder/President	67%
Steven^*^^~^	18	M	International club	Leadership Team	83%
Jesus^*^	17	M	Cyber security club	Founder/Vice President	100%
Roberto^~^	16	M	Star wars club	Member	100%
Stefana^~^	18	F	Tabletop game club	President	67%

* *indicates a student who was interviewed as part of the study.*

~ *indicates a student who was observed during club activities*.

### Measures

A survey, the *Special Education Teacher Extracurricular Survey*, was designed for this study and distributed using Qualtrics. The first page of the survey included a description of the study and an opt-in for participants to provide informed consent. The survey included 24 multiple-choice, Likert-type and open-ended items. Example survey items contained in the survey include “How often do you promote opportunities to get involved in clubs to students with autism”, “In your experience, how often do students with autism, or their parents, inquire about extracurricular opportunities at school?”, and “Which of the following do you perceive as barriers to students with autism participating in extracurricular activities?” This survey is available for reference in Appendix A.

An observation protocol originally designed by Carter and colleagues to examine the impact of peer support arrangements in general education classrooms [see (25)] was modified to gather specific information on the participation and social interaction of students with autism occurring in clubs. This *Club Observation Sheet* was scored utilizing a combination of partial-interval recording and momentary time-sampling (a 30-s partial-interval observational periods followed by a 20-s recording window, momentary time-sampling at the :50 second mark, and field notes recorded in the final 10 s). Twenty 1-min intervals were scored during each observation session. During each interval, researchers scored the occurrence of the following: social behaviors (verbal or non-verbal interaction between individuals) (a) from the target student with autism to a peer, (b) from the target student with autism to a staff member, (c) from a peer to the target student with autism, and (d) from a staff member to the target student with autism. Researchers also recorded whether the target student initiated (defined as “to facilitate the beginning of”) an interaction during each interval. The focus of the club activity and whether the activity being facilitated within each interval supported any of the 4Cs was also recorded. The 4Cs were defined as: creativity (CRE), using imagination or formulating original ideas; critical thinking (CT), analysis and/or evaluation in order to form a judgement or solve a problem; collaboration (COL), two or more individuals working together to produce or create something; and communication (COM), the verbal exchange of information, ideas or feelings. The format of the club activity [full group (FULL), small groups (SM), individual work time (IN), or no structured activity (NST)] was marked in the field notes.

Finally, in-person, semi-structured interviews with students were conducted using a fourteen-item interview protocol designed to gather the perspective of students with autism related to the value of club participation. Sample questions included “Has your case manager ever talked to you about clubs or activities on campus?” and “In this club, do you talk to your peers about the activities you are doing?” The interview protocol is available for reference in Appendix B.

### Procedures

Data collection took place concurrently in the spring semester of Year 3 of the CSESA RCT. The *Special Education Teacher Extracurricular Survey* was distributed via Qualtrics to 97 special educators serving on CSESA teams at their school sites, and 34 completed surveys were received (response rate: 35%). The survey was sent twice; an initial invitation to participate, followed by a reminder 3 weeks later.

Once student participants were identified and assent was received (for either their participation in the interview, club observations, neither, or both), the CSESA site coordinators assisted the research team with scheduling. Interviews with the students were conducted at the school site during a preferred time and were audio recorded and transcribed for data analysis. Staff advisors/leaders of the clubs in which students participated were identified by the site coordinators. They were contacted and connected with the research team. Once approved, three club meeting observations were conducted per student. Observational sessions began 5 min after the start of the club meeting and were 20 min in length.

### Data Analysis

Observational and survey data were transferred to IBM SPSS Statistics (Version 25) prior to analysis. Interview responses, once transcribed, were coded in Dedoose using the phases of thematic analysis outlined by Braun and Clarke (26). A directed approach to qualitative content analysis was used to identify themes across participants and settings regarding the experiences of students with autism in extracurricular high school clubs, with codes generated and organized in alignment with the study research questions in a top-down approach. These qualitative interview data were compared and contrasted with quantitative frequency data gained through the survey responses from special educators and observations of the students in club environments. Themes emerging from these multiple sources of data were triangulated and interpreted comprehensively.

#### Interobserver Agreement

Three M.A. degree candidates who had prior experience working in school settings with students with autism were trained as second observers. Training was conducted prior to data collection using video recordings of students engaged in group activities and a coding manual designed to systemize scoring using the *Club Observation Sheet*. Each rater was required to score 80% or higher inter-rater agreement with the lead researcher in two consecutive training videos prior to conducting any observations. Interobserver agreement (IOA) was scored item by item using the following formula: the number of agreements divided by the number of agreements plus the number of disagreements, multiplied by 100 (27). Interobserver agreement was an overall mean of 98.6%, with a range from 96.4% (Stefana) to 99.6% (Courtney). The average IOA ranged from 95% (for social interactions from target student to peers and peers to the target student) to 100% (for social interactions from target student to special educators, critical thinking, and club format).

## Results

The intent of this investigation was to explore the experiences of high school students with autism in extracurricular club settings and to determine whether club environments are contexts in which 21st century skills, including communication, are currently being practiced. Thematic analysis of student interviews resulted in the emergence of seven broad themes related to their participation in clubs, including (a) the opportunity to practice skills, (b) growing their interest/knowledge in particular subject area, (c) broadening their perspectives, (d) developing as leaders, (e) serving the community, (f) having fun, and (g) making friends/socializing. Results are organized by research question (RQ) and presented with information about the data sources used to address the question, followed by triangulation and interpretation of the data.

### RQ 1. What Types of Clubs Are Students With Autism Involved In?

The *Special Education Teacher Extracurricular Survey* and interview data were used to investigate this question. Special educators were asked to report the specific activities that interested their students with autism (Table 2), and most frequently reported video gaming (18), table games (9), animation (7), dance (5), sports (5), food (4), movies (4), and computers/technology (4).

**Table 2:** Club content of interest reported by special educator survey respondents.

**Club content**	**Listed as interest by special educators**	**Mentioned as interest by students w/ autism during interview(s)**
Academic league		
Anime club	x	x
Art club	x	
Associated student body		
Brother 2 brother		
California scholarship federation		
Chess club		
Christian club		x
Club fandom	x	x
Club INSPIRE (service learning)		
Dance club	x	
Drama club	x	
Environmental club		
French club		
French honors society		
Gay straight alliance		
Gender equality club		
Girls learn international		x
Global language and leadership club		x
Hip-hop club		
Impact club		
International club		x
Key club		
Service learning		
Math club		
National honor society		
Newspaper club		
Outdoor outreach club		
Pirate pics		
Raspberry pi club	x	x
Reader's society	x	x
Russian club		
Saturday scholars		
Skateboarding club		
Speech and debate		
Surf club		
Tabletop game club	x	x
TED Ed club		
Tennis club		
Theater club	x	
UNICEF club		
Video game club	x	x

Many of the clubs that the students who were interviewed/observed as part of this study are aligned with the special educators' reported areas of interest for students with autism. Luke was president and founder of a video game club, and also participated in an animation (anime) club; Tyrese was founder and president of a movie club; and Jesus was vice president of a cyber security club and participated in a robotics club. Steven was a leader in an “international” club, which centered largely on discussing and sampling foods from cultures around the world. Students were also asked in their interviews whether there were other clubs that they were interested in; these responses which overlapped with the areas of interest reported by special educators are also indicated in Table 2.

### RQ 2. How Do Students Get Involved in Extracurricular Clubs on High School Campuses, and Are There Barriers to Participation for Students With Autism?

Several avenues pertaining to the ways students get involved in clubs in high schools were identified in special educator survey responses and student interviews. The six students interviewed all attended different schools, but five of them expressed that the main opportunity to explore club offerings at their schools is during the annual “club day,” an event held near the beginning of each academic year where clubs set up booths to promote their activities and sign-up new members. The club day event was how Luke became aware of, and involved in, his school's anime club. Later, when he decided to start his own video game club, he explained, “I set up our booth at club day, and we actually got a surprisingly large amount of signatures. It was a good start to the club to know that other kids at school were interested.” Courtney and Steven mentioned that club activities were promoted in announcements made through the speaker systems at their schools, and Harry and Jesus explained that sometimes posters are placed around the school to promote various club events. Notably, Courtney, Steven, and Jesus each expressed that a staff member at their school was integral in their process of joining their club, reaching out directly to them or to their family to encourage them to participate.

Special educators were asked to indicate on a Likert scale how often they promoted club participation to students with autism. A substantial number of special educators (76%) reported that they often or almost always did, with only 3 percent of special educators indicated that they never or seldomly promoted club activities to students with autism. In response to the statement “I consider it part of my job to facilitate extracurricular activity participation for my students with autism,” 82% percent of special educators indicated that they either “strongly agree” or “agree.” When asked how often students with autism (or their parents) inquired about extracurriculars at school, however, 74% percent of special educators responded that they only “sometimes” or “seldom” did. Interestingly, 30% of special educators “disagree” with the statement “I am aware of the club offerings available at my school site.”

Regarding barriers to participation, students were asked during interviews whether anything has ever prevented them from joining a club of interest (see Table 3). Only one major barrier- lack of awareness of the existence of the club- was identified by the students, and all six who were interviewed mentioned this barrier. Inconsistent with student reports, special educators were asked to select from a list of potential barriers which they perceived to limit club participation for students with autism and the most commonly selected deterrents were lack of interest, social challenges, and lack of transportation. They were also given the chance to name additional barriers not included on the list, and several reported concern for lack of sufficient staff to support students with autism in extracurricular settings.

**Table 3:** Perceived barriers to club participation.

**Barrier**	**Special educators (*N* = 34)**
Students are not interested in participating	26
Social challenges that might arise in extracurricular settings deter students from joining	19
Transportation is not available if student participates in extracurricular activities	22
Students do not know how to go about joining activities	17
Students are unaware of extracurricular opportunities that exist at school ^*^	11
Activities offered at my school don't match student interests	12
Student schedule doesn't allow for participation	8
Students may be bullied in extracurricular settings	5
Extracurricular activities are not welcoming to students with autism	3

* *indicates a barrier that was also mentioned by students interviewed in the study*.

### RQ3. Are There Opportunities for Social Interaction With Peers During Extracurricular Clubs for Students With Autism, and If So, to What Extent?

Special educator survey responses, student interviews, and observations of student club meetings were used to address this question. Responding to the statement “extracurricular activities offer valuable opportunities for all students to develop effective social communication skills,” 94% of special educators indicated that they “strongly agree” or “agree.” In response to the statement “my students with autism have experienced improved or increased peer interactions since joining a school-sponsored extracurricular activity,” 75% of special educators reported that they “strongly agree” or “agree.”

In order to explore the opportunities that students have to interact socially during club meetings, 18 separate observations were conducted during normally scheduled club meetings. Table 4 provides a complete summary of the observational data collected. Overall, the students with autism engaged in social interaction with others in 62% of recorded intervals, ranging from 43% (Courtney) to 92% (Luke). The students initiated communication with others in an average of 46% of recorded intervals; individual averages ranged from 15% (Courtney) to 68% per student (Luke), indicating that students with autism within club environments have regular opportunities to engage with peers and to practice social skills.

**Table 4:** Summary of observational data across study.

	**Courtney**	**Luke**	**Tyrese**	**Steven**	**Roberto**	**Stefana**
**Measure**	**Girls learn**	**Video**	**Movie**	**International**	**Star wars club**	**Tabletop**
	**international**	**game club**	**club**	**club**	**club**	**game club**
**Social Interactions (%)**						
Any initiations	15 (0–35)	68 (50–80)	37 (30–45)	60 (55–65)	32 (25–35)	63 (60–70)
Any interactions	43 (5–70)	92 (85–95)	45 (35–55)	67 (55–80)	52 (40–65)	72 (65–80)
Student to peer	25 (0–50)	67 (60–95)	18 (5–30)	60 (55–70)	45 (30–65)	67 (65–70)
Student to staff	12 (0–20)	12 (0–20)	32 (25–40)	33 (25–40)	10 (0–20)	27 (25–30)
Peer to student	35 (5–60)	75 (60–90)	18 (5–30)	50 (45–60)	47 (35–70)	65 (55–75)
Staff to student	22 (5–35)	13 (0–25)	28 (20–35)	28 (20–35)	13 (10–20)	25 (25–25)
**21st Century Skill Focus (%)**						
Creativity	45 (10–100)	5 (0–10)	3 (0–10)	23 (20–30)	32 (0–80)	22 (0–50)
Critical Thinking	23 (0–45)	3 (0–10)	0 (0–0)	5 (0–15)	27 (0–80)	43 (15–65)
Collaboration	62 (40–90)	43 (10–80)	8 (0–15)	32 (15–60)	25 (15–45)	57 (15–100)
Communication	93 (90–100)	80 (60–95)	37 (20–45)	88 (70–100)	65 (30–100)	72 (45–100)
**Club Format (%)**						
Full Group	77 (30–100)	95 (85–100)	33 (0–100)	88 (85–95)	62 (0–95)	0 (0–0)
Small Groups (>7)	23 (0–30)	0 (0–0)	67 (0–100)	7 (0–15)	33 (0–100)	97 (90–100)
Individual work	0 (0–0)	0 (0–0)	0 (0–0)	0 (0–0)	0 (0–0)	0 (0–0)
No structured activity	0 (0–0)	5 (0–15)	0 (0–0)	5 (0–15)	5 (0–15)	3 (0–10)

*The figures represent M (range). Each club was observed three times, with IOA measured once each*.

Student opinions varied on whether club settings helped to facilitate peer interaction. While some students admitted in interviews to relying heavily on club meetings for their socialization with peers (Courtney, Stefana, Roberto,), others did not express that engaging with peers was a priority for them—whether in club settings or otherwise. Their responses were unanimous, however, in expressing the belief that clubs were an easier context in which to engage socially with peers than were other contexts at school (in class, at lunch, etc.).

As Tyrese explained,

…in my opinion it's easier to make friends or engage with other kids [in a club setting]. [In answer to interviewer's query:] Because you're not forced to be together or hang out. You are choosing to. And if you're having fun with the club, you can keep going to the club and can just keep meeting new friends. It makes you really committed to the club.

### RQ 4. Do Participants in High School Clubs Have the Opportunity to Work on the 4Cs (Communication, Collaboration, Creativity, and Critical Thinking) in Club Environments?

Special educator surveys, student interviews, and observational data were utilized to explore this question. In their interviews, students were asked about opportunities within their clubs to be creative, work collaboratively, and solve problems—along with opportunities they had to communicate with others, as discussed in the previous section. Their responses indicated that there were clear, natural opportunities for using the skills of creativity, critical thinking, collaboration, and communication within club contexts. Harry explained that collaboration is essential within his stage crew club:

A lot of collaboration is just natural in the work. You've got to get certain things done by a certain time and day. And usually, we fall behind, and then we have to find a way to get things done quickly. Or, like sometimes, we'll get requests for projects really last minute.

Steven described the need for creativity, collaboration, and communication in international club:

The problems that we've had as a club are usually money, they're financial. We have our treasurer for that, you know, counting up how much money we've spent over the year. And we'll decide what to do if we need more money in order to do an activity or plan an event that we want to host. We've done some candy fundraisers I think. And what we do every fall, for the homecoming carnival, is we sell street tacos. We have a booth for that.

Jesus, who participates in cyber security club, echoed the responses of Steven and Harry when describing the opportunities that his club gave him to work on 21st century skills. He explained that, although the technical aspect of cyber security might lead people to believe it is a solo endeavor, the opportunity to work on cyber security projects in a competitive club setting also demands communication, critical thinking, creativity and collaboration—all of the 4Cs.

Special educators were asked if they believe that clubs give students with and without autism opportunities “to develop 21st century skills (i.e., communication, collaboration, creativity, critical thinking), and 97% indicated that they “strongly agree” or “agree” that they do. These stakeholder reports were confirmed through observations of the club environments. Table 4 provides the percentage of recorded intervals in which the activities taking place within each club setting directly supported the 4C skill areas. Averages and range for opportunities to practice these skills across all six observed club settings, from least to most frequently observed, were as follows: critical thinking (*M* = 17%, range: 0–43%), creativity (*M* = 22%, range: 3–45%), collaboration (*M* = 38%, range: 8–62%), and communication (*M* = 73%, range: 37–93%).

### RQ 5. What Do Students With Autism Report About Their Experiences in High School Clubs, Including the Experiences They Value, the Aspects They Identify as Challenges, and Their Preferences for Peer Interaction?

While the bulk of the data to answer this question came from student interviews, survey responses from special educators were considered in order to provide context for student responses. All six students interviewed considered club meetings a highlight of their week, a way for them to have fun during the school day, and as a way to foster peer relationships. Three of the students reported that they have friends in their club with whom they spend time outside of club meetings. All six students reported that they enjoyed interacting with like-minded peers within club settings.

As discussed above, special educators reported that they believe clubs offer valuable learning and social experiences for students. The students interviewed asserted their belief that clubs provide a sense of shared community and purpose. Courtney observed:

Clubs are really important. I like how we're, like, talking about different problems around the world, like how girls from different places should receive good educations. Like any other girl in America. […] And we learn about what other girls around the world experience. It's something everyone should be more aware of.

Interestingly, four of the students expressed interest in turning their passion for their club activity into a future career path. Students seemed to value this feature of club participation as unique in their overall school experience. Jesus and Tyrese expressly stated that they believe their club activities (cyber security and movie clubs, respectively) would be instrumental in getting them admitted into post-secondary programs aimed at careers in those industries. Interviewees also mentioned real-life challenges they had to handle as part of their club roles. Jesus mentioned learning lessons as a leader of his club, stating that the hardest aspect was:

…the leadership position, because you get to realize it's a lot of responsibility. Like, we had some meetings recently and the president, I think he was in charge of the calendar, but he didn't update it, and since I was one of the leaders, I don't know... I guess the club advisor was kind of disappointed, because I was also a leader, so I was supposed to be doing that, like maybe helping him a little bit more. It's a big responsibility.

Luke reflected on the fact that, as a person with autism, the challenges that he experienced as club president may not mirror those experienced by other club presidents: “...just all the strengths and weaknesses that I have, I feel like they would be lessened if I did not have autism. I might be a better communicator, or have a shorter attention span or less attention to detail, who knows?”

## Discussion

Despite the exploratory nature of this research, there were findings that support the benefits of participation in extracurricular clubs for high school students with autism. It is clear that the high schools in this study offered a wide variety of extracurricular club opportunities, and that these opportunities aligned with the reported interests of students with autism. The overlap between the club topics of interest reported by both special educators and students with autism indicates the potential for the clubs currently offered at high schools to be attractive spaces to these students.

Special educators tended to view non-participation in clubs as a result of lack of interest rather than lack of awareness; however, they did report that they considered it part of their job to encourage students to participate in extracurricular activities Students find out about and get involved in clubs through a variety of channels, including club day events and school announcements, but school staff also play an important role in promoting clubs to students with autism (who may not seek out these opportunities independently). Whether or not these invitations are extended to them is largely dependent on the school staff members with whom they have relationships, and the nature of those relationships.

Special educators indicated that they consider clubs valuable for promoting social interaction with peers and building 21st century skills, and this value was confirmed through observations and interviews. Clubs provide rich and frequent opportunities for students to engage with one another, and the group activities that are included in club meetings provide frequent, applied opportunities for students to practice 21st century skills. The students with autism weren't only in attendance at their respective club meetings– they were active. They had regular opportunities to practice the 4Cs throughout the observed club gatherings and spent significant portions of the meetings interacting with their peers. The wide-ranging topics of observed club meetings suggests that clubs can, and in some cases do, provide opportunities for participants to practice skills that the name of the club alone may not convey (for example, participants in the Girls Learn International club had the opportunity to practice the skill of communication in 93% of observed intervals). Even club topics that are not generally considered “educational” offer valuable opportunities for students to practice the 4Cs. Overall, during observed club meetings, the students with autism engaged in social interaction with others in 62% of recorded intervals, which is a much higher rate of interaction than previous studies have indicated happens in general education classroom settings (25). All students reported that they enjoyed interacting with peers within club settings and that they found it easier to engage with peers in club environments.

Beyond socialization, students reported valuing clubs for a variety of reasons, including for opportunities to practice skills or participate in activities not offered in content area classes. An unexpected finding was the amount of leadership experience that clubs provided to students with autism. Several participants reported that they founded their own clubs or were serving in leadership roles, and others mentioned that they had formulated post-secondary career goals related to their club topics. Perhaps Steven said it best, when he reflected that “people who join clubs, they can become very passionate about an issue, and they can make a difference.”

### Study Limitations

A limitation to this pilot is the modest number of both student and special educator participants. The survey sent to special educators generated a response rate of 35% and a relatively small sample size (*N* = 34). A broader distribution of this survey would have allowed for increased generalizability of the results. Additionally, increasing the number of students interviewed and observed in their school clubs, and carrying out observations over a longer period of time, may have allowed for a more complete understanding of the school club experience for students with autism. It would also have been useful to include in the sample students with autism who do not participate in clubs, to gain perspective on barriers to participation not experienced by those who are participants. Ultimately, the level of access to participants in this study was both facilitated and limited by the CSESA project. It is possible that there were CSESA student participants who participated in clubs at their school that the site coordinators were unaware of, and if so, these students were not included. Due to the fact that the sample for this study was taken from the greater CSESA sample and recruitment for the present study was largely facilitated by the individual site coordinators, relationships with many of the eventual participants in this study were already established, but there may also have been study fatigue.

### Implications for Practice

The study results suggest several recommendations for school personnel in high schools. It is important that school sites consider the opportunities that all students, and particularly those with disabilities, have to explore and learn about extracurricular opportunities on campus. This opportunity is ultimately an issue of equity and access. Special education departments should ensure that their staff are well-versed in the school's club offerings so that special educators can confidently discuss club opportunities with students with autism and their parents. Results from this study indicated a lack of clarity on the part of high school special educators about the value of extracurricular clubs. Special educators consider promoting these opportunities a part of their jobs, but how? Is it clear that all students are welcome? School and district-level policy implications could be drawn from these data to plan for more comprehensively promoting extracurricular club participation to all students. Explaining the additional skill development opportunities present in club settings, regardless of the topic, might also encourage families to promote them to their children. Clubs could be discussed throughout the year, not just on “Club Day,” as students become more comfortable with their teachers and as their interests develop and change.

Mastery of 21st century skills is crucial for all students to be successful, whether in postsecondary education or in the workforce. Therefore, the adjustment of club activities and/or the embedding of specific strategies to target these vital 21st century skills within club environments might be effective as a group-wide intervention rather than one designed solely for the benefit of students with autism. Connecting these 21^st^ century skills to club participation has already begun in the field of secondary special education, including in the school-based PEERS® curriculum (14). To further support this vision, professional development for school staff members related to 21st century skill development might prove valuable. Special educators could also collaborate with other members of the school community though professional learning communities (PLCs) in order to brainstorm strategies for helping clubs serve the student populations at their schools in new and innovative ways. This collaboration might also help special educators gain confidence that clubs at their school sites are appropriate settings for their students with autism.

### Further Research

As the expectations for graduating students become more rigorous and the number of students with autism educated in public schools grows, a larger and more geographically diverse study should be conducted in order to determine whether or not the results found in this study are regionally unique. It would also be beneficial to extend this research to other extracurricular settings (sports, religious groups, scout groups, etc.) in order to determine whether similar opportunities for building 21st century skills exist in those contexts. Continuing to search for meaningful environments in which youth can develop 21st century skills will only grow in importance as the field of special education grapples with how best to support the one-third of capable young adults who are “disconnected” after high school (i.e., have no job or postsecondary education) (6). Finally, an intervention study focused on embedding strategies to improve peer interaction and 21st century skills in club contexts would further benefit not only students with autism, but all students.

## Data Availability Statement

The original contributions presented in the study are included in the article/supplementary materials, further inquiries can be directed to the corresponding author.

## Ethics Statement

The studies involving human participants were reviewed and approved by San Diego State University. Written informed consent to participate in this study was provided by the participants' legal guardian/next of kin. Written informed consent was obtained from the individual(s), and minor(s)' legal guardian/next of kin, for the publication of any potentially identifiable images or data included in this article.

## Author Contributions

LH and BK provided research and writing support. All authors of this manuscript worked collaboratively to design, carry out, and author the study.

## Funding

Preparation of this paper was supported by Grant No. R324C120006 from the Institute of Education Sciences, U.S. Department of Education. Opinions expressed are not necessarily those of the funding agency.

## Conflict of Interest

The authors declare that the research was conducted in the absence of any commercial or financial relationships that could be construed as a potential conflict of interest.

## Publisher's Note

All claims expressed in this article are solely those of the authors and do not necessarily represent those of their affiliated organizations, or those of the publisher, the editors and the reviewers. Any product that may be evaluated in this article, or claim that may be made by its manufacturer, is not guaranteed or endorsed by the publisher.
