# Supplementary material for: Exploring Extracurricular Clubs for Building Social Competence of Students With Autism
Source: Front Psychiatry. 2022 Mar 23;13:840294. doi: 10.3389/fpsyt.2022.840294 (PMC8984242; doi:10.3389/fpsyt.2022.840294)
Supplement: Supplementary file 1 [file Data_Sheet_1.docx]

**Appendix A**

**Special Education Teacher Extracurricular Survey**

1) What is your gender?

1. Female
2. Male
3. Non-binary /other identified
4. Prefer to self-describe
5. Prefer not to say

2) In what areas do you hold a teaching credential?

3) How long have you been teaching (round to nearest year)?

4) How long have you been teaching at this school (round to nearest year)?

5) How many years of overall experience do you have teaching students with autism?

6) How many students do you have on your caseload this year with autism?

7) Do you teach any students with autism who spend the majority of their time in general education settings and who will graduate with a traditional high school diploma?

**For purposes of this study, a school-sponsored extracurricular activity is defined as:**

*“An activity centered around a specific topic or activity of interest, which meets outside of class time and regularly throughout the school year, and is approved by the school administration. Participation is voluntary, although students may need to meet certain requirements to participate.”* ***Please do not include friendship-oriented groups, such as buddy clubs, or disability-specific activities, such as Special Olympics, in your responses.***

8) How many students with autism on your caseload participate in school-sponsored extracurricular activities?

9) In your experience, how often are students with autism involved in school clubs?

1. Almost always
2. Often
3. Sometimes
4. Seldom
5. Never

10) How often do you, as a case manager, promote opportunities for students with autism to get involved in clubs?

1. Almost always
2. Often
3. Sometimes
4. Seldom
5. Never

11) In your experience, how often do students with autism or their parents, inquire about extracurricular opportunities at school?

a) Almost always

b) Often

1. Sometimes
2. Seldom
3. Never

12) Have you served as an advisor or support to a school-sponsored extracurricular activity anytime in the past 3 years?

1. Yes, as an advisor
2. Yes, as a support to a specific student/group of students
3. No

13) If yes, what was the activity?

**How strongly do you agree with the following statements?**

14) I’m aware of the full range of clubs and extracurricular activities that are offered at the school where I teach, and when these clubs meet.

1. Strongly Agree
2. Agree
3. Undecided
4. Disagree
5. Strongly Disagree

15) Extracurricular activities on this campus are welcoming to students with autism.

1. Strongly Agree
2. Agree
3. Undecided
4. Disagree
5. Strongly Disagree

16) There are barriers to students with autism participating in clubs on campus.

1. Strongly Agree
2. Agree
3. Undecided
4. Disagree
5. Strongly Disagree

17) Which of the following, if any, do you perceive as barriers to students with autism participating in extracurricular activities on campus? (check all that apply)

1. Students are not interested in participating
2. Activities offered at my school don’t match student interests
3. Students are unaware of extracurricular opportunities that exist at school
4. Students do not know how to go about joining activities
5. Extracurricular activities are not welcoming to students with autism
6. Social challenges that might arise in extracurricular settings deter students from joining
7. Students may be bullied in extracurricular settings
8. Student schedule doesn’t allow for participation
9. Transportation is not available if student participates in extracurricular activities
10. Other: _____________________________________

**How strongly do you agree with the following statements?**

18) Extracurricular activities offer valuable opportunities for students with autism to develop effective social communication skills.

1. Strongly Agree
2. Agree
3. Undecided
4. Disagree
5. Strongly Disagree

19) Extracurricular activities offer valuable opportunities for students with autism to develop 21^st^ century skills (i.e. collaboration, creativity, critical thinking)?

1. Strongly Agree
2. Agree
3. Undecided
4. Disagree
5. Strongly Disagree

20) Participation in extracurricular activities can help students develop important skills that are not explicitly taught in traditional high school classes.

1. Strongly Agree
2. Agree
3. Undecided
4. Disagree
5. Strongly Disagree

21) I consider it part of my job to facilitate extracurricular activity participation for my students with autism.

1. Strongly Agree
2. Agree
3. Undecided
4. Disagree
5. Strongly Disagree

22) My students with autism have experienced improved or increased peer interactions since joining a school-sponsored extracurricular activity.

1. Strongly Agree
2. Agree
3. Undecided
4. Disagree
5. Strongly Disagree

23) Which specific extracurricular activities tend to interest your students with autism (club content, type of activities, etc.)?

24) What do you view as the most important reasons for students with autism, particularly those students who are diploma-bound and included in general education, to participate in extracurricular activities at school?
