# Supplementary material for: Exploring Extracurricular Clubs for Building Social Competence of Students With Autism
Source: Front Psychiatry. 2022 Mar 23;13:840294. doi: 10.3389/fpsyt.2022.840294 (PMC8984242; doi:10.3389/fpsyt.2022.840294)
Supplement: Supplementary file 2 [file Data_Sheet_2.docx]

**Appendix B**

**Semi-Structured Interview Protocol for Student Club Participants**

{*PP*}= *Potential Probes*

**(1) Would you describe for me the types of things that you like to do in your free time?**{*PP*} What do you do when you’re not at school?
 What do you find fun or enjoyable?
 Do you have a favorite topic or activity?

**(2) How did you find out about the club?**

{*PP*} What is the process of joining a club on campus like?

**(3) Did you choose to join the ­­­__________ ­­­­­­­­­­­­­­­club?**

{*PP*} What about the topic or content area appealed to you?

Were you already interested in this topic before joining the club?
Did anybody tell you to join this activity?

**(4) Do you like participating in this club?**

**(5) What is the best part about being in this club?**
{*PP*} Do you think other students with autism should participate in clubs? Why or why not?

**(6) Has your case manager ever talked to you about clubs or activities on campus?**

**(7) What is the hardest part about being in a school club?**

{*PP*} Can you think of a solution to this challenge?

**(8) Do you ever skip club meetings?**

*{PP*} If so, why?

**(9) Do you feel like there is anything stopping you from joining clubs that you want to join?**

**(10) Do you think there is anything that can, or should, be done to address these barriers to joining clubs?**

{*PP*} Is there anything the school or club advisors could do to help?
 Is there anything that the special education team or case managers could do to help? **(11) In this club, do you talk to your peers about the activities you are doing?**

{*PP*} If no, would you be willing to do this as part of the club’s activities?
 Do you work on collaborative projects as part of the club?
 Do you work together create or come up with new ideas in this club?
 Do you solve problems in this club?
 IF YES, *{pp}* Can you give me an example of this?

**(12) Do you have any friends in the club you’re in?**

{*PP*} Are there any particular peers that you spend time with or talk to during clubs?
 Is it easier to engage socially in clubs verses other settings, such as class or at lunch?

Do you ever hang out with club members outside of the club? Outside of school?

**(13) Are you involved in any other clubs besides ____________?**

{*PP*} How long have you been a member?

**(14) If you could start a school club, what would it be?**

{*PP*} Do you think other students would be interested in this club, too?

How would your club be different than other clubs offered at your high school?
